# The relationship between synovitis quantified by an ultrasound 7-joint inflammation score and physical disability in rheumatoid arthritis – a cohort study

**DOI:** 10.1186/s13075-016-1208-6

**Published:** 2017-01-13

**Authors:** Jakub Závada, Petra Hánová, Jana Hurňáková, Lenka Szczuková, Michal Uher, Šárka Forejtová, Martin Klein, Herman Mann, Marta Olejárová, Olga Růžičková, Olga Šléglová, Karel Hejduk, Karel Pavelka

**Affiliations:** 1 Institute of Rheumatology, Prague, and Department of Rheumatology, First Faculty of Medicine, Charles University, Na Slupi 4, 12850 Prague, Czech Republic; 2 Institute of Biostatistics and Analyses, Faculty of Medicine, Masaryk University, Brno, Czech Republic

**Keywords:** Rheumatoid arthritis, Ultrasonography, Synovitis, Health assessment questionnaire

## Abstract

**Background:**

Restoring normal physical functioning is a major therapeutic aim in the management of rheumatoid arthritis (RA). It is unknown, whether the extent of synovial inflammation quantified by musculoskeletal ultrasound (US) can predict current or future capacity for physical functioning. To answer this question we investigated the longitudinal relationship between physical function assessed by the health assessment questionnaire (HAQ) and the German 7-joint ultrasound score (US7S) in a prospective cohort of patients with RA.

**Methods:**

Patients with RA (*n* = 185 (46 with incident and 139 with prevalent disease) were followed for 30.9 ± 9.1 months. Baseline and annual assessments comprised the disease activity score in 28 joints (DAS28), HAQ and US7S. The US7S includes semiquantitative measurements of synovitis assessed by greyscale (GS) and power Doppler (PD) in seven joints of the clinically dominant hand and foot, which are then aggregated in PD and GS synovitis sum-scores (PDsynSS and GSsynSS). A linear mixed-effect model was used to assess the longitudinal relationship between GSsynSS, PDsynSS and HAQ. We used standard and time-lag models to explore the association between HAQ, and GSsynSS, PDsynSS and DAS28 measured at the same time or at the previous visit 12 months ago, respectively.

**Results:**

When the standard model was applied, in univariate analyses HAQ score was positively associated with GSsynSS and PDsynSS with β coefficients significantly higher in incident than in prevalent disease. In multivariate analysis both synSSs were individually no longer significant predictors of HAQ score. When using the time-lag model, after adjustment for the previous DAS28 or HAQ score, both PDsynSS and GSsynSS were significantly and negatively associated with the current HAQ.

**Conclusions:**

US7 PD and GS synovitis sum-scores alone were positively associated with current functional status reflected by the HAQ in patients with RA, and this relationship was stronger in patients with early disease. When combined with the DAS28 or HAQ, US7 PD and GS synovitis sum-scores were predictive of the change in HAQ score over one year.

**Electronic supplementary material:**

The online version of this article (doi:10.1186/s13075-016-1208-6) contains supplementary material, which is available to authorized users.

## Background

Rheumatoid arthritis (RA) may lead to substantial functional impairment caused by the combined effect of potentially reversible inflammatory activity and irreversible destructive articular and periarticular damage [1–4]. The main objective of RA management is to prevent and/or reverse the decline of physical functioning by aggressive control of disease activity and thus halting the structural progression.

The currently recommended tight control and treat-to-target RA management strategies are guided by close monitoring of disease activity using composite indexes such as the disease activity score in 28 joints (DAS28), simplified disease activity index (SDAI) or clinical disease activity score (CDAI) [5]. However, clinical disease activity indexes also have several limitations [6]: clinical examination may be poorly reproducible [7, 8] and may fail to capture subclinical synovitis associated with progressive joint damage [9, 10]), especially in the feet [11–13]. Disease activity scores may be falsely increased in some patients without evidence of synovitis due to fibromyalgia [14].

An important question is whether these limitations of conventional disease activity scores may be alleviated by complementing clinical examination with musculoskeletal ultrasound (US), which has been shown to be more sensitive [15–18] and reliable [19–21] in detecting synovitis compared to physical examination. The German US7 score (US7S) has been shown to be a feasible, reliable and sensitive tool for the US examination of inflammatory joint activity in RA [22–24]. The US7S includes seven joints of the clinically dominant hand and foot, consists of five additive sum-scores for synovitis (syn) and tenosynovitis (ten) assessed by greyscale (GS) and power-Doppler (PD), and an erosions score (ES). It is unknown whether an US joint inflammation score may contribute additional prognostic information on measures of functional impairment beyond the standard clinical indexes.

In our longitudinal prospective cohort study, we have systematically performed repeated clinical and US assessments of joint inflammation (using the DAS28 and US7S) and physical function evaluations (using the health assessment questionnaire (HAQ)) in patients with early RA and established RA. The main objectives of the present analysis were to examine the ability of US7S synovitis sum scores (PDsynSS, GSsynSS) to predict the HAQ score measured concurrently or 12 months later, either alone or as an adjunct to the DAS28 (or previous HAQ).

## Methods

### Patients

Patients with early RA (*n* = 46) newly started on therapy with conventional synthetic disease-modifying drugs (csDMARDs) or glucocorticoids (incident cohort), and 139 patients with established RA (prevalent cohort) were asked to participate in this this study. Diagnosis of RA was based on the American College of Rheumatology/European League against Rheumatism (EULAR) 2010 classification criteria for RA [25]. The patient characteristics are provided in Table 1. All patients were recruited from the outpatient rheumatology clinic at the Institute of Rheumatology in Prague and were followed longitudinally according to a predefined protocol. Informed consent was obtained from all patients before entry into the study. Patients on biological DMARDs were not included in the study, because they are followed separately in the Czech biologics ATTRA registry. During the observation period, patients were routinely treated by their rheumatologist. The study was approved by the Ethics Committee of the Institute of Rheumatology in Prague (Reference number 3560/2010) and was conducted according to the guidelines of the Declaration of Helsinki.

**Table 1:** Baseline characteristics

	Total (*N* = 185)	Incident RA (*N* = 46)	Prevalent RA (*N* = 139)	*P* value
Female (%)	76.8	69.6	79.1	0.066
Age (years)	55.2 (14.0)	51.7 (12.6)	56.4 (14.2)	<0.001*
Disease duration (years)	6.3 (7.7)	0.8 (0.7)	8.1 (8.1)	<0.001*
Symptom duration (years)	7.3 (7.7)	1.5 (1.8)	9.2 (8.0)	<0.001*
BMI	26.7 (4.5)	27.5 (5.2)	26.4 (4.3)	0.020*
RF+ (%)	47.2	43.5	48.5	0.466
ACPA+ (%)	62.9	58.7	64.3	0.952
CRP (mg/l)	7.6 (9.0)	10.5 (12.6)	6.6 (7.1)	0.354
DAS28-CRP	3.7 (1.5)	4.0 (1.5)	3.6 (1.5)	0.192
GSsynSS	6.91 (6.39)	7.70 (5.62)	6.65 (6.61)	0.255
PDsynSS	4.02 (5.20)	5.24 (5.95)	3.61 (4.87)	0.438
GStenSS	0.66 (1.15)	0.78 (1.18)	0.63 (1.14)	0.842
PDtenSS	0.68 (1.63)	0.80 (1.75)	0.63 (1.59)	0.402
Erosions score	1.18 (2.10)	0.46 (1.50)	1.42 (2.21)	<0.001*
HAQ	0.77 (0.73)	0.88 (0.73)	0.74 (0.73)	0.896
Oral glucocorticoids (%)	43.8	41.3	44.6	0.607
csDMARDs (%)	87.0	69.6	92.8	<0.001*

Values are mean (SD) if not stated otherwise. *RA* rheumatoid arthritis, *BM*I body mass index, *RF* rheumatoid factor, *ACP*A anti-citrullinated peptide antibodies, *CRP* C-reactive protein, *DAS28* disease activity score in 28 joints, *GS* greyscale, *PD* power Doppler, *syn* synovitis, *ten* tenosynovitis, *ES* erosions score, *SS* sum-score, *HAQ* health assessment questionnaire, *csDMARDS* conventional synthetic disease-modifying anti-rheumatic drugs. *Significant at *p* < 0.05

### Outcome measurements

The same measures of clinical assessment and US imaging were applied at baseline and every 6 months during the follow-up period. Physical function was assessed by the HAQ [26] at baseline and then annually. For the purpose of this analysis only clinical and US data collected concurrently with the HAQ every 12 months were used. Demographic and anthropometric data collection, and data on rheumatoid factor (RF), and anti-citrullinated protein antibody (ACPA) testing were performed at baseline. Tender and swollen joint counts were carried out on 28 joints by an experienced nurse, in accordance with the EULAR recommendations [27]. C-reactive protein (CRP) was measured in a local laboratory using turbidimetry (Beckman Coulter, CA, USA). For the purpose of this analysis, we have used the DAS28 with CRP (DAS28-CRP) as the main outcome measure of clinical activity.

### Ultrasound imaging

The US examinations were performed by eight clinicians who have undergone intermediate to advanced US training. The results of a practical exercise published elsewhere [28] showed moderate interobserver agreement for assessment of PD synovitis, substantial agreement for assessment of GS synovitis, and excellent intrareader agreement. We used the Esaote Mylab 60 equipment (Esaote S.p.A., Genova, Italy) and a linear transducer with an 18-MHz frequency. The power Doppler sensitivity was pre-set (pulse repetition frequency 750 Hz, wall filter 3/5, persistence 4/16, colour flow mapping frequency 7.1 MHz), and no adjustments of the Doppler parameters were allowed.

The patients were examined according to the German US7 score in the following seven joint areas: wrist, second and third metacarpophalangeal (MCP) and second and third proximal interphalangeal (PIP) and second and fifth metatarsophalangeal (MTP) joints of the hand and foot that clinically was more affected [22]. We used a modification of the original German US7 [29], which is currently used and endorsed by the authors of both the original and modified versions (personal communication with M. Backhaus). In contrast to the original US7, which examines synovitis of the MCP and PIP joints in GS only from the palmar view; we assessed synovitis in GS in this area using both the palmar and the dorsal view. Further, while the original German US7 assesses tenosynovitis/paratenonitis on the second and third finger both from the palmar and dorsal aspect, we assessed tenosynovitis only from the palmar aspect. Synovitis in the GS was scored semiquantitatively (0 = absence, 1 = mild, 2 = moderate, 3 = severe synovitis), as follows: grade 1 = a small hypoechoic/anechoic line beneath the joint capsule; grade 2 = the joint capsule elevated parallel to the joint area; and grade 3 = strong distension of the joint capsule [30].

Synovitis and tenosynovitis were classified semiquantitatively by PD, as follows: grade 0 = no intraarticular color signal; grade 1 = up to three single colour signals or two single signals and one confluent signal in the intraarticular area; grade 2 = greater than grade 1 to <50% of the intraarticular area filled with colour signals; and grade 3 = ≥50% of the intraarticular area filled with colour signals. Tenosynovitis in the GS was registered as absent (0) or present (1). An overall GS and PD signal score was calculated as the sum of GS synovitis, PD synovitis and GS tenosynovitis and PD tenosynovitis with the range of scores of 0–39 for GS synovitis, 0–39 for PD synovitis, 0–5 for GS tenosynovitis and 0–15 for PD tenosynovitis. The ultrasonographers were unaware of each patient’s clinical examination and laboratory findings.

### Statistical analysis

Differences in baseline variables between the included patients with early (*n* = 46) and established (*n* = 139) RA (incident and prevalent cohorts, respectively) were examined by the Mann-Whitney test (continuous variables) or Fisher’s exact test (categorical variables). A linear mixed effects model (LMM) was used to study the longitudinal relationship between the clinical measures of disease activity (DAS28-CRP), US inflammatory score (German US7 scores sum-scores for GS and PD synovitis, GS and PD tenosynovitis, and erosions) as explanatory variables, and physical function (HAQ score) as a dependent variable. Age, sex, rheumatoid factor (RF) and anti-CCP status, body mass index (BMI) and disease duration were also introduced in one of the multivariate models as covariates. All the subsequently described analytical models used in our study were pre-planned. To explore the influence of current disease activity (assessed either clinically or by US) on the current HAQ score, we used the standard (“current”) model. To explore the impact of previous disease activity (assessed either clinically or by US) on the current HAQ score we used a “time-lag” model. In the time-lag model the covariates measured at the previous visit (12 months before) are related to the outcome variable assessed at the current visit. Also, the previous HAQ (assessed at the previous visit 12 months earlier) was added to the model (i.e. the first-order autoregression) to model change in the HAQ score rather than absolute HAQ scores. The proportion of variability in the HAQ scores explained by given covariates or models was assessed using *R* ^2^ and compared to each other using analysis of variance (ANOVA).

Participants with missing data on baseline characteristics and parameters of disease activity were included in the analysis by using multiple imputations using the Markov chain Monte Carlo method with 30 imputations obtained after 20 iterations. Variables included in imputation models were all baseline covariates and parameters of disease activity (variables with missing data were imputed and used as predictors, and variables with complete data were used as predictors only). IBM SPSS Statistics version 22 (SPSS Inc., Chicago, IL, USA) was used for the analyses. All *P* values equal to or less than 0.05 were considered statistically significant.

## Results

### Descriptive analyses

The baseline characteristics of the whole cohort, and the incident and prevalent cohorts, are shown in Table 1. Most of the patients were female (77%), mean age was 55 years, 47% were RF-positive and 63% ACPA-positive. At the baseline examination, 87% of the patients were taking conventional csDMARDs, and 44% oral glucocorticoids. The patients with early RA were younger (52 vs. 56 years; *P* < 0.001), had significantly shorter disease duration than patients with established disease (0.9 vs. 8.1 years; *P* < 0.001), had significantly lower erosions scores (0.5 vs. 1.4; *P* < 0.001), and were less frequently taking csDMARDs (69 vs. 93%; *P* < 0.001). Mean (SD) length of follow up was 30.9 (9.1) months.

In the whole cohort, the average HAQ scores were relatively stable over the 3-year follow-up period, while both clinical and US parameters of disease activity improved (Additional file 1). However, the evolution of functional and activity measures in time was somewhat different in the incident vs. prevalent cohorts (Additional files 2 and 3). While the mean HAQ scores had dropped during the first year of observation in patients with early RA, there was a steady increasing trend in HAQ scores in patients with established disease. Also, there was a more pronounced decrease in both clinical and US activity parameters in the incident cohort than in the prevalent cohort since the baseline visit.

### Cross-sectional associations between ultrasound inflammatory score and physical function (standard or current model)

In univariate analyses (Table 2) the HAQ score was positively associated with GSsyn, PDsyn, PDten and GSten US7 sum-scores with β coefficients significantly higher in patients with incident than in patients with prevalent disease, respectively. However, the proportion of variability of the HAQ score explained by GSsynSS, PDsynSS, GStenSS and PDtenSS was relatively small (*R* ^2^ of 2–4% for US7S as compared to *R* ^2^ of 41% for DAS28-CRP), and the erosions score was not correlated with HAQ score at all. In a multivariate analysis that included conventional demographic, immunological and clinical variables (Table 3), female gender, age and DAS28-CRP were all significantly and positively associated with HAQ score, while US7 sum-scores were individually no longer significant predictors of HAQ score and the *R* ^2^ of the whole model improved only marginally after the addition of US7 items (from 44.7 to 45.9; *P* < 0.001 for improvement in *R* ^2^).

**Table 2:** Standard (current) model

Predictor	RA	β (95% CI)	*P* value^*^	*P* value^**^	Percent variability explained - *R* ^2^
DAS28-CRP	all	0.186 (0.157; 0.216)	*<0.001*		41.3
	i	0.234 (0.174; 0.295)	*<0.001*	0.072	
	p	0.171 (0.137; 0.204)	*<0.001*		
GSsynSS	all	0.018 (0.011; 0.026)	*<0.001*		3.2
	i	0.035 (0.020; 0.050)	*<0.001*	*0.013*	
	p	0.013 (0.004; 0.022)	*0.003*		
PDsynSS	all	0.025 (0.015; 0.035)	*<0.001*		3.6
	i	0.038 (0.021; 0.055)	*<0.001*	0.056	
	p	0.018 (0.006; 0.030)	*0.004*		
GStenSS	all	0.048 (0.004; 0.092)	*0.033*		2.4
	i	0.123 (0.039; 0.207)	*0.004*	*0.038*	
	p	0.019 (-0.032; 0.071)	0.460		
PDtenSS	all	0.051 (0.019; 0.082)	*0.002*		2.8
	i	0.100 (0.039; 0.162)	*0.001*	0.064	
	p	0.033 (-0.003; 0.070)	0.073		
ES	all	0.007 (-0.022; 0.036)	0.632		0.3
	i	-0.024 (-0.102; 0.054)	0.549	0.390	
	p	0.013 (-0.019; 0.045)	0.416		

Univariate analyses and interaction with incident (i) or prevalent (p) rheumatoid arthritis (RA). Predicted variable is current health assessment questionnaire (HAQ) score, univariate predictors are current disease activity score in 28 joints using C-reactive protein (DAS28-CRP), sum-scores (SS) for greyscale (GS) and power Doppler (PD) synovitis, GS and PD tenosynovitis (ten), and erosions score (ES). ^*^ *P* value of significance of given β. ^**^ *P* value of significance of difference between incident and prevalent RA. *P*-values higlighted in italics are significant at *p* < 0.05

**Table 3:** Standard (current) model: multivariate analyses

Predictor	Model without US-7		Model with US-7	
	β (95% CI)	*P* value	β (95% CI)	*P* value
Female	0.211 (0.045; 0.376)	*0.013*	0.208 (0.044; 0.372)	*0.013*
Age (years)	0.149 (0.097; 0.201)	*<0.001*	0.147 (0.096; 0.199)	*<0.001*
BMI	0.008 (-0.007; 0.024)	0.276	0.008 (-0.007; 0.023)	0.291
RF+ or ACPA+	0.029 (-0.115; 0.172)	0.696	0.029 (-0.114; 0.172)	0.691
Prevalent vs. incident RA	-0.048 (-0.209; 0.113)	0.558	-0.050 (-0.212; 0.111)	0.541
DAS28-CRP	0.184 (0.155; 0.213)	*<0.001*	0.202 (0.166; 0.238)	*<0.001*
GSsynSS	-		-0.003 (-0.014; 0.008)	0.595
PDsynSS	-		-0.001 (-0.015; 0.014)	0.916
GStenSS	-		-0.062 (-0.129; 0.006)	0.073
PDtenSS	-		0.018 (-0.032; 0.068)	0.487
Erosions score	-		0.005 (-0.022; 0.032)	0.720
*R* ^2^ (% variability explained)	44.7		45.9	
Improved *R* ^2^	-		1.1	*<0.001* *

Predicted variable is current health assessment questionnaire (HAQ) score. Comparison of models based on demographic, clinical and immunological parameters with or without the German 7-joint ultrasound score (US7) sum-scores (SS). *P value of significance for improvement of prediction. *BMI* body mass index, *RF* rheumatoid factor, *ACPA* anti-citrullinated peptide antibodies, GS greyscale, *PD* power Doppler, *syn* synovitis, *ten* tenosynovitis, *ES* erosions score. *P*-values higlighted in italics are significant at *p* < 0.05

### Longitudinal associations between ultrasound inflammatory score and physical function (time-lag model)

When applying a time-lag model, in univariate analyses only previous HAQ score and DAS28-CRP, but not the US7 sum-score, was predictive of current HAQ score measured 12 months apart (Table 4). However, in multivariate analyses (Table 5) after adjustment for previous DAS28 and/or previous HAQ score, both previous PDsynSS and GSsynSS were significantly and inversely associated with the current HAQ score. Previous PDsynSS remained a statistically significant predictor of current HAQ score, even in a more extensive multivariate model after adjustment for the most important conventional demographic, immunological and clinical variables (Table 6), and the percent variability of HAQ score explained by this model was substantially improved by the addition of the US7S sum-scores (from 32 to 39%, *P* < 0.001).

**Table 4:** Time-lag model

Predictor	RA	β (95% CI)	*P* value*	*P* value**	Percent variability explained - *R* ^2^
Previous DAS28-CRP	all	0.102 (0.062; 0.143)	*<0.001*		29.0
	i	0.072 (-0.008; 0.152)	0.079	0.353	
	p	0.115 (0.069; 0.161)	*<0.001*		
Previous GSsynSS	all	0.003 (-0.006; 0.012)	0.530		7.4
	i	0.000 (-0.019; 0.019)	1.000	0.713	
	p	0.004 (-0.007; 0.015)	0.465		
Previous PDsynSS	all	-0.003 (-0.015; 0.009)	0.605		0.4
	i	-0.001 (-0.021; 0.019)	0.924	0.816	
	p	-0.004 (-0.018; 0.011)	0.595		
Previous GStenSS	all	0.006 (-0.044; 0.056)	0.822		0.7
	i	0.005 (-0.089; 0.099)	0.922	0.972	
	p	0.007 (-0.053; 0.066)	0.825		
Previous PDtenSS	all	-0.005 (-0.041; 0.032)	0.801		0.5
	i	-0.019 (-0.092; 0.054)	0.606	0.655	
	p	0.000 (-0.042; 0.042)	1.000		
Previous ES	all	0.028 (-0.007; 0.062)	0.118		0.4
	i	-0.043 (-0.158; 0.072)	0.462	0.216	
	p	0.033 (-0.004; 0.070)	0.081		
Previous HAQ	all	0.740 (0.676; 0.803)	*<0.001*		63.5
	i	0.627 (0.496; 0.759)	*<0.001*	*0.050*	
	p	0.775 (0.705; 0.846)	*<0.001*		

Univariate analyses and interaction with incident (i) or prevalent (p) rheumatoid arthritis (RA). Predicted variable is current HAQ score, univariate predictors are previous disease activity score in 28 joints using C-reactive protein (DAS28-CRP), sum-scores for greyscale (GS) and power Doppler (PD) synovitis (syn), GS and PD tenosynovitis (ten), and erosions score (ES). *P value of significance of given β. **P value of significance of difference between incident and prevalent RA. *P*-values higlighted in italics are significant at *p* < 0.05

**Table 5:** Time-lag model: multivariate analyses

Analysis number	Predictor	β (95% CI)	*P* value	Percent variability explained by the model (*R* ^2^)
1	Previous HAQ	0.762 (0.698; 0.827)	*<0.001*	64.8
	Previous PDsynSS	-0.017 (-0.027; -0.007)	*0.001*	
2	Previous PDsynSS	-0.025 (-0.039; -0.011)	*<0.001*	32.8
	Previous DAS28	0.156 (0.109; 0.203)	*<0.001*	
3	Previous HAQ	0.703 (0.620; 0.786)	*<0.001*	65.4
	Previous PDsynSS	-0.022 (-0.034; -0.011)	*<0.001*	
	Previous DAS28	0.050 (0.005; 0.096)	*0.029*	
4	Previous HAQ	0.754 (0.690; 0.819)	*<0.001*	64.2
	Previous GSsynSS	-0.009 (-0.017; -0.001)	*0.024*	
5	Previous GSsynSS	-0.012 (-0.023; -0.001)	*0.036*	32.5
	Previous DAS28	0.135 (0.088; 0.183)	*<0.001*	
6	Previous HAQ	0.707 (0.623; 0.792)	*<0.001*	64.5
	Previous GSsynSS	-0.013 (-0.022; -0.004)	*0.006*	
	Previous DAS28	0.039 (-0.007; 0.085)	0.096	

Predicted variable is current health assessment questionnaire (HAQ) score. Predictors used in the six analyses are either previous HAQ score or disease activity score in 28 joints (DAS28) or both, and previous power Doppler (PD) synovitis (SS) or greyscale (GS) syn sum-score (SS) (previous = measured 12 months ago. ES = erosions score. *P*-values higlighted in italics are significant at *p* <  0.05

**Table 6:** Time-lag model: multivariate analysis, extended model

Predictor	Model without US-7		Model with US-7	
	β (95% CI)	*P* value	β (95% CI)	*P* value
Female	0.182 (-0.007; 0.370)	0.059	0.167 (-0.012; 0.347)	0.068
Age (years)	0.172 (0.113; 0.231)	*<0.001*	0.169 (0.113; 0.226)	*<0.001*
BMI	0.009 (-0.009; 0.026)	0.316	0.007 (-0.010; 0.024)	0.405
RF+ or ACPA+	0.060 (-0.103; 0.224)	0.471	0.052 (-0.106; 0.210)	0.517
Prevalent vs. incident RA	0.026 (-0.156; 0.209)	0.779	0.010 (-0.165; 0.186)	0.910
Previous DAS28-CRP	0.100 (0.061; 0.140)	*<0.001*	0.161 (0.113; 0.208)	*<0.001*
Previous GSsynSS			-0.004 (-0.019; 0.010)	0.563
Previous PDsynSS			-0.021 (-0.040; -0.002)	*0.035*
Previous GStenSS			0.000 (-0.085; 0.085)	0.997
Previous PDtenSS			-0.015 (-0.078; 0.048)	0.643
Previous erosions score			0.012 (-0.022; 0.046)	0.484
*R* ^2^ (% variability explained)	32.3		39.1	
Improved *R* ^2^			6.8	*<0.001* *

Predicted variable is current health assessment questionnaire (HAQ) score. Comparison of models based on baseline demographic, anthropometric and immunological parameters, and previous disease activity score in 28 joints (DAS28) with or without previous German 7-joint ultrasound score (US7) sum-scores (SS) (previous = measured 12 months ago). *P value of significance for improvement of prediction. *RA* rheumatoid arthritis, *BMI* body mass index, *RF* rheumatoid factor, *ACPA* anti-citrullinated peptide antibodies, *GS* greyscale, *PD* power Doppler, *syn* synovitis, *ten* tenosynovitis. *P*-values higlighted in italics are significant at *p* < 0.05

### Additional analyses

One possible explanation for why previous higher values of PDsynSS are associated (in the multivariate model) with improvement of functional status between the previous and current visit, may be the fact that treatment of RA was escalated between the previous and current visit. To examine this we conducted several analyses with PDsynSS (and DAS28, or HAQ) as exploratory variables and escalation of therapy (defined as new/increased use of glucocorticoids or DMARDS within 6 months after the index visit) as a dependent variable (see Additional files 4 and 5). Although in univariate analyses (Additional file 4) all parameters (PDsynSS, DAS28, and HAQ) were positively associated with escalation of therapy within the next 6 months, after adjustment for previous HAQ score or DAS28, previous PDsynSS was no longer an independent predictor of escalation of therapy (Additional file 5).

## Discussion

Preservation of physical function represents a fundamental long‐term outcome for patients with RA. To our knowledge, this is the first study that systematically examines the longitudinal relationship between an US joint inflammation score and physical function in patients with RA. Several previous studies have informed us on the relationship between the conventional clinical, radiological and laboratory measures of disease activity and HAQ evolution [1–4], but we have lacked deeper knowledge on the impact of synovitis detected by US on the HAQ score.

Musculoskeletal US is increasingly being used to detect and monitor joint inflammation in RA, in both clinical practice and clinical research. Indeed, both GSUS and PDUS have been shown to have greater sensitivity for detecting synovitis as compared with physical examination [9, 10], are more reliable [14–16] and detect response to therapy more rapidly in comparison to various clinical scores [31–34]. Because US examination of all joints and tendons that can be affected in RA would be extremely time consuming, several reduced joint scores for assessing US joint inflammation have been developed. Concentration on a small number of active joint regions reduces examination time, but still retains most of the quantitative information compared to the extended joint scores [35–39]. It is important to stress that we have mainly been examining the relationship between the HAQ score (as a dependent variable), and the DAS28 and US7S synovitis sum-scores (as explanatory variables). This does not mean that we have been comparing only the additive role of ultrasound examination to clinical joint counts. DAS28-CRP is a construct based on a 28-joint count of swollen and tender joints, patient global assessment and CRP. While most patient-reported outcome measures tend to be highly correlated, the representation of the patient global assessment and the number of tender joints in the DAS28 helps to explain a substantial part of the variability in HAQ score. US7S synovitis sum-scores reflect synovitis in the five joints of the clinically dominant hand (wrist, MCP II, III, PIPI II, III) and two joints of the foot (MTP II and V), but synovitis of the wrist is assessed from the dorsal, volar and ulnar aspects, synovitis of the MCP and PIP joints from the dorsal and volar aspects, MTP V from the dorsal and lateral aspects, and MTP II only from the dorsal aspects. Hence, the joints of the hand (11 joint areas) are represented much more than the joints of the foot (3 joint areas) in the sum-scores for PD and GS synovitis.

Using LLM analysis, we applied two main models - a standard (current) model and a time-lag model - in a cohort of patients with RA that included both patients with early and with established RA. While the standard model reflects mainly the cross-sectional relationship between covariates and predicted variables collected at the same time, the time-lag model allows for more longitudinal interpretation of the data. When applying the standard model, the PD and GS synovitis sum-scores were significantly positively correlated with current HAQ score and the relative impact of US-detected synovitis was twice as high in patients with early RA as compared to those with established disease. A similar trend was seen in the tenosynovitis score, while the erosions score was not significantly associated with current HAQ score. This finding is in accordance with other studies showing that the reversible part of the HAQ related to disease activity is higher in early RA, while prolonged duration of the disease increases the part of the HAQ related to irreversible damage caused by structural joint damage [1–4, 40–42].

The magnitude of the effect of the individual US7 sum-scores on HAQ score in univariate analyses was relatively minor (explained percent variability in the HAQ score was in the range of 3 − 5%) and became statistically insignificant when adjustments were made for the conventional clinical and anthropometric variables, although the performance of the whole multivariate model was still slightly better when all US7S sum-scores were entered into the model. These findings suggest that at least in this population of patients with RA, the ultrasound measures included in the US7S were only weakly associated with overall physical functioning as reflected by the HAQ score. This may be partly related to the design of the HAQ itself - although this instrument is capable of detecting small but meaningful changes in function in individual patients [43], it is dominated by effects on large joints such as the hips, knees and shoulders (which are not represented in the US7S), and is relatively insensitive in detecting changes in, for example, hand function, which is mainly represented in the US7S [44, 45].

The results of the time-lag model revealed a somewhat different perspective. Although the univariate analyses relating previous US7 sum-scores to current HAQ score using the time-lag model were non-significant, both PDsynSS and GSsynSS appeared to be significant predictors of current HAQ after adjustment for the previous DAS28 or HAQ score, and previous PDsynSS contributed additional information on current HAQ even in the more extensive multivariate model. This apparent discordance may be explained by the fact that by adding previous HAQ (or DAS28, which is highly correlated with HAQ) into the multivariate model creates an autoregressive model that mainly predicts the change in HAQ score.

Somewhat counterintuitively, higher PDUS and GSUS synovitis scores were longitudinally (when using the time-lag model after adjustment for previous DAS28 or HAQ) associated with improvement (not deterioration) of HAQ. This probably reflects the ability of PDUS and GSUS to discriminate between the activity-driven reversible part of the HAQ and the irreversible part of the HAQ caused by structural damage. The additional analysis on the role of clinical and US-based measures to determine escalation of therapy suggested that the main driver of change was mainly the clinical score, and not the US7S. Although the treating physicians were allowed to see the results of the US assessment, they were not actively encouraged to apply them in their decision-making, because the role of the US-based inflammatory score for treat-to-target strategies was not established. This may mean that the part of the HAQ associated with the impact of synovitis detected by PDUS or GSUS is reversible (either because of change in therapy, or because of spontaneous fluctuation in disease activity), and does not necessarily contribute - at least in the time-frame of one year - to the irreversible part of the HAQ caused by ensuing structural damage. Because patients with longer follow up contributed more data, we compared baseline characteristics between groups of patients who completed one, two or three annual follow-up visits and found no significant trends or differences (Additional file 6).

Our study has several limitations. In our analyses we treated the US7 sum-scores as a linear continuum, equalizing the effect of three joints with a PD or GS signal of 1 to a single joint with a PD or GS signal of 3. This assumption may not be entirely correct, as shown by others [46]. However the primary goal of this analysis was to assess the relationship of one specific US score (US7S) either alone or in combination with conventional clinical tools, and hence we used the US7 sum-scores as designed by its authors. The fact that US examination of patients was performed by different observers may also be a limitation of this study, as US is still considered to be operator-dependent. In order to verify interobserver and intraobserver agreement among arthro-sonographers in this project, we performed a reliability study, which was published elsewhere [28], and showed moderate to excellent results.

Our study also has several strengths. To our knowledge, a true longitudinal analysis of the relationship between an ultrasound inflammation score and physical function, after adjustment for clinical measures of disease activity, has never been published. The most important advantage of longitudinal data analysis, including the LLM method, is that all available data are used, which increases the power to detect subtle relationships. We have analyzed data from 185 patients overall (46 with incident and 139 with prevalent disease) and 522 follow-up visits.

## Conclusions

In conclusion, we found that US7 PD and GS synovitis sum-scores alone were positively associated with current functional status reflected by the HAQ score in RA patients, and this relationship was stronger in patients with early disease. However, they contributed little additional information on the current HAQ score, beyond standard clinical measures. When combined with the DAS28, or HAQ, US7 PD and GS synovitis sum-scores were predictive of the change in HAQ score over one year. Based on the multivariate analyses of the time-lag model and taking into the account the results of univariate analyses of the standard model we may assume that US7S sum-scores for synovitis were associated mainly with the reversible activity-related component of HAQ, and may thus help to identify patients with higher chance of functional improvement. Although the interpretation of the results is not straightforward, our study adds further argument to caution against indiscriminate use of more aggressive therapeutic approaches (with the aim of preventing irreversible damage) based on information from the US inflammatory score, beyond standard clinical activity indexes.

## Additional files

Additional file 1: Descriptive characteristics of the cohort over time^1^. (DOCX 22 kb)
Additional file 2: Descriptive characteristics of the incident cohort only over time^1^. (DOCX 21 kb)
Additional file 3: Descriptive characteristics of the prevalent cohort only over time^1^. (DOCX 22 kb)
Additional file 4: Prediction of escalation of therapy - univariate analyses (with and without stratification for incident vs. prevalent disease, and low vs. moderate to high disease activity). (DOCX 21 kb)
Additional file 5: Prediction of escalation of therapy - multivariate analyses. (DOCX 24 kb)
Additional file 6: Comparison of baseline variables between groups of patients with one, two and three years of follow up. (DOCX 24 kb)

## Abbreviations

ACPA: anti-citrullinated protein antibody
ANOVA: analysis of variance
BMI: body mass index
CDAI: clinical disease activity Score
CRP: C-reactive protein
csDMARDs: conventional synthetic disease modifying drugs
DAS28: disease activity score in 28 joints
ES: erosions score
EULAR: European League against Rheumatism
GS: greyscale
GSsynSS: greyscale synovitis sum-score
HAQ: health assessment questionnaire
LMM: linear mixed-effects model
MCP: metacarpophalangeal
MTP: metacarpophalangeal
PD: power-Doppler
PDsynSS: power-Doppler synovitis sum-score
PIP: proximal interphalangeal
RA: rheumatoid arthritis
RF: rheumatoid factor
SDAI: simplified disease activity score
Syn: synovitis
Ten: tenosynovitis
US: ultrasound
US7S: German 7-joint ultrasound score
