# Supplementary material for: The relationship between synovitis quantified by an ultrasound 7-joint inflammation score and physical disability in rheumatoid arthritis – a cohort study
Source: Arthritis Res Ther. 2017 Jan 13;19:5. doi: 10.1186/s13075-016-1208-6 (PMC5237153; doi:10.1186/s13075-016-1208-6)
Supplement: Additional file 1: — Descriptive characteristics of the cohort over time1. (DOCX 22 kb) [file 13075_2016_1208_MOESM1_ESM.docx]

**Additional file 1**

Descriptive characteristics of the cohort over time^1^

| **Time point of follow-up** | **Month 0** | **Month 12** | **Month 24** | **Month 36** |
| --- | --- | --- | --- | --- |
| **No. of patients** | 185 | 185 | 98 | 54 |
| **HAQ (scale 1-3)** |  |  |  |  |
| Mean ± SD score | 0.77 (0.73) | 0.77 (0.68) | 0.81 (0.66) | 0.82 (0.62) |
| Median (IQR) | 0.62 (0.12; 1.25) | 0.63 (0.12; 1.25) | 0.63 (0.25; 1.38) | 0.88 (0.25; 1.25) |
| Max/Min | 2.88/0.00 | 2.75/0.00 | 2.94/0.00 | 2.64/0.00 |
| Δ vs. M0^*^(Mean ± SD) | 0.00 (0.00) | -0.01 (0.49) | -0.03 (0.54) | 0.00 (0.57) |
| Δ vs. M-12^**^(Mean ± SD) | 0.00 (0.00) | -0.01 (0.49) | -0.04 (0.39) | 0.00 (0.33) |
| **GSsynSS** |  |  |  |  |
| Mean ± SD score | 6.91 (6.39) | 4.24 (4.56) | 3.85 (3.61) | 3.52 (3.34) |
| Median (IQR) | 5 (2; 9) | 3 (1; 6) | 3 (1; 6) | 3 (1; 5) |
| Max/Min | 29/0 | 25/0 | 22/0 | 16/0 |
| **GStenSS** |  |  |  |  |
| Mean ± SD score | 0.66 (1.15) | 0.29 (0.70) | 0.21 (0.47) | 0.16 (0.49) |
| Median (IQR) | 0 (0; 1) | 0 (0; 0) | 0 (0; 0) | 0 (0; 0) |
| Max/Min | 5/0 | 5/0 | 2/0 | 3/0 |
| **PDsynSS** |  |  |  |  |
| Mean ± SD score | 4.02 (5.20) | 2.51 (3.25) | 2.38 (2.97) | 2.11 (2.59) |
| Median (IQR) | 2 (1; 5) | 1 (0; 4) | 1 (0; 4) | 1 (0; 4) |
| Max/Min | 28/0 | 18/0 | 15/0 | 11/0 |
| **PDtenSS** |  |  |  |  |
| Mean ± SD score | 0.68 (1.63) | 0.32 (1.12) | 0.33 (0.77) | 0.24 (0.89) |
| Median (IQR) | 0 (0; 0) | 0 (0; 0) | 0 (0; 0) | 0 (0; 0) |
| Max/Min | 9/0 | 10/0 | 4/0 | 6/0 |
| **ES** |  |  |  |  |
| Mean ± SD score | 1.18 (2.10) | 1.19 (1.90) | 1.00 (1.68) | 1.32 (1.92) |
| Median (IQR) | 0 (0; 2) | 0 (0; 2) | 0 (0; 1) | 1 (0; 2) |
| Max/Min | 12/0 | 9/0 | 8/0 | 8/0 |
| **DAS28** |  |  |  |  |
| No. of patients | 185 | 185 | 98 | 54 |
| Mean ± SD score | 3.67 (1.51) | 3.06 (1.19) | 2.93 (1.30) | 2.84 (1.21) |
| Median (IQR) | 3.75 (2.36; 4.82) | 2.94 (2.03; 3.85) | 2.68 (1.80; 3.67) | 2.65 (1.76; 3.59) |
| Max/Min | 7.92/1.12 | 7.13/1.09 | 7.52/1.07 | 6.50/1.12 |

^1^ see separate tables for incident and prevalent cohorts in additional files 1-2.
*Change compared to baseline **Change compared to previous measurement

GS=gray scale; PD=power doppler; syn = synovitis, ten=tenosynovitis, ES= erosions score; SS=sum-score
