# Supplementary material for: The relationship between synovitis quantified by an ultrasound 7-joint inflammation score and physical disability in rheumatoid arthritis – a cohort study
Source: Arthritis Res Ther. 2017 Jan 13;19:5. doi: 10.1186/s13075-016-1208-6 (PMC5237153; doi:10.1186/s13075-016-1208-6)
Supplement: Additional file 2: — Descriptive characteristics of the incident cohort only over time1. (DOCX 21 kb) [file 13075_2016_1208_MOESM2_ESM.docx]

**Additional file 2**

Descriptive characteristics of the ***incident cohort*** *only* over time^1^

|  | M0 | M12 | M24 | M36 |
| --- | --- | --- | --- | --- |
| **HAQ (scale 1-3)** |  |  |  |  |
| No. of patients | 46 | 46 | 23 | 10 |
| Mean ± SD score | 0.88 (0.73) | 0.68 (0.66) | 0.79 (0.60) | 0.62 (0.52) |
| Median (IQR) | 0.88 (0.12; 1.50) | 0.45 (0.12; 1.13) | 0.63 (0.38; 1.50) | 0.63 (0.00; 1.13) |
| Max/Min | 2.50/0.00 | 2.46/0.00 | 2.00/0.00 | 1.38/0.00 |
| Δ vs. M0^*^(Mean ± SD) | 0.00 (0.00) | -0.20 (0.58) | -0.14 (0.54) | -0.14 (0.74) |
| Δ vs. M-12^**^(Mean ± SD) | 0.00 (0.00) | -0.20 (0.58) | 0.03 (0.36) | 0.04 (0.28) |
| **GSsynSS** |  |  |  |  |
| Mean ± SD score | 7.70 (5.62) | 2.87 (3.10) | 2.96 (2.96) | 2.26 (2.74) |
| Median (IQR) | 7 (3; 13) | 2 (1; 4) | 2 (0; 4) | 1 (0; 5) |
| Max/Min | 20/0 | 17/0 | 10/0 | 12/0 |
| **GStenSS** |  |  |  |  |
| Mean ± SD score | 0.78 (1.18) | 0.18 (0.53) | 0.30 (0.69) | 0.01 (0.11) |
| Median (IQR) | 0 (0; 1) | 0 (0; 0) | 0 (0; 0) | 0 (0; 0) |
| Max/Min | 5/0 | 3/0 | 2/0 | 1/0 |
| **PDsynSS** |  |  |  |  |
| Mean ± SD score | 5.24 (5.95) | 1.58 (2.42) | 2.00 (2.29) | 1.13 (1.94) |
| Median (IQR) | 3 (1; 8) | 1 (0; 2) | 1 (0; 3) | 0 (0; 1) |
| Max/Min | 24/0 | 13/0 | 8/0 | 9/0 |
| **PDtenSS** |  |  |  |  |
| Mean ± SD score | 0.80 (1.75) | 0.10 (0.36) | 0.35 (0.96) | 0.04 (0.20) |
| Median (IQR) | 0 (0; 1) | 0 (0; 0) | 0 (0; 0) | 0 (0; 0) |
| Max/Min | 7/0 | 2/0 | 4/0 | 1/0 |
| **ES** |  |  |  |  |
| Mean ± SD score | 0.46 (1.50) | 0.26 (0.85) | 0.22 (0.66) | 0.79 (1.55) |
| Median (IQR) | 0 (0; 0) | 0 (0; 0) | 0 (0; 0) | 0 (0; 1) |
| Max/Min | 8/0 | 5/0 | 3/0 | 5/0 |
| **DAS28** |  |  |  |  |
| No. of patients | 46 | 46 | 23 | 10 |
| Mean ± SD score | 3.99 (1.48) | 2.96 (1.11) | 2.98 (1.12) | 3.00 (1.32) |
| Median (IQR) | 4.20 (2.53; 5.07) | 2.91 (1.90; 3.65) | 3.07 (1.81; 3.91) | 2.85 (1.71; 3.66) |
| Max/Min | 6.06/1.18 | 5.72/1.09 | 4.80/1.07 | 5.59/1.12 |

GS=gray scale; PD=power doppler; syn = synovitis, ten=tenosynovitis, ES= erosions score; SS=sum-score
