# Supplementary material for: The relationship between synovitis quantified by an ultrasound 7-joint inflammation score and physical disability in rheumatoid arthritis – a cohort study
Source: Arthritis Res Ther. 2017 Jan 13;19:5. doi: 10.1186/s13075-016-1208-6 (PMC5237153; doi:10.1186/s13075-016-1208-6)
Supplement: Additional file 3: — Descriptive characteristics of the prevalent cohort only over time1. (DOCX 22 kb) [file 13075_2016_1208_MOESM3_ESM.docx]

**Additional file 3**

Descriptive characteristics of the ***prevalent cohort*** *only* over time^1^

|  | M0 | M12 | M24 | M36 |
| --- | --- | --- | --- | --- |
| **HAQ (scale 1-3)** |  |  |  |  |
| No. of patients | 139 | 139 | 75 | 44 |
| Mean ± SD score | 0.74 (0.73) | 0.79 (0.68) | 0.82 (0.68) | 0.87 (0.63) |
| Median (IQR) | 0.62 (0.12; 1.12) | 0.75 (0.13; 1.25) | 0.75 (0.13; 1.38) | 0.94 (0.25; 1.31) |
| Max/Min | 2.88/0.00 | 2.75/0.00 | 2.94/0.00 | 2.64/0.00 |
| Δ vs. M0^*^(Mean ± SD) | 0.00 (0.00) | 0.06 (0.43) | 0.01 (0.54) | 0.03 (0.51) |
| Δ vs. M-12^**^(Mean ± SD) | 0.00 (0.00) | 0.06 (0.43) | -0.06 (0.39) | 0.00 (0.34) |
| **GSsynSS** |  |  |  |  |
| Mean ± SD score | 6.65 (6.61) | 4.70 (4.86) | 4.12 (3.75) | 3.80 (3.40) |
| Median (IQR) | 5 (2; 9) | 3 (1; 6) | 3 (1; 6) | 3 (1; 5) |
| Max/Min | 29/0 | 25/0 | 22/0 | 16/0 |
| **GStenSS** |  |  |  |  |
| Mean ± SD score | 0.63 (1.14) | 0.32 (0.75) | 0.17 (0.38) | 0.19 (0.54) |
| Median (IQR) | 0 (0; 1) | 0 (0; 0) | 0 (0; 0) | 0 (0; 0) |
| Max/Min | 5/0 | 5/0 | 1/0 | 3/0 |
| **PDsynSS** |  |  |  |  |
| Mean ± SD score | 3.61 (4.87) | 2.82 (3.43) | 2.50 (3.14) | 2.33 (2.67) |
| Median (IQR) | 2 (0; 5) | 2 (0; 4) | 1 (0; 4) | 2 (0; 4) |
| Max/Min | 28/0 | 18/0 | 15/0 | 11/0 |
| **PDtenSS** |  |  |  |  |
| Mean ± SD score | 0.63 (1.59) | 0.40 (1.26) | 0.33 (0.71) | 0.28 (0.97) |
| Median (IQR) | 0 (0; 0) | 0 (0; 0) | 0 (0; 0) | 0 (0; 0) |
| Max/Min | 9/0 | 10/0 | 3/0 | 6/0 |
| **ES** |  |  |  |  |
| Mean ± SD score | 1.42 (2.21) | 1.50 (2.05) | 1.24 (1.82) | 1.45 (1.98) |
| Median (IQR) | 0 (0; 2) | 1 (0; 2) | 0 (0; 2) | 1 (0; 2) |
| Max/Min | 12/0 | 9/0 | 8/0 | 8/0 |
| **DAS28** |  |  |  |  |
| No. of patients | 139 | 139 | 75 | 44 |
| Mean ± SD score | 3.56 (1.50) | 3.09 (1.22) | 2.91 (1.35) | 2.80 (1.18) |
| Median (IQR) | 3.48 (2.28; 4.62) | 3.04 (2.10; 3.99) | 2.64 (1.80; 3.63) | 2.60 (1.76; 3.56) |
| Max/Min | 7.92/1.12 | 7.13/1.10 | 7.52/1.14 | 6.50/1.22 |

GS=gray scale; PD=power doppler; syn = synovitis, ten=tenosynovitis, ES= erosions score; SS=sum-score
