# Supplementary material for: The relationship between synovitis quantified by an ultrasound 7-joint inflammation score and physical disability in rheumatoid arthritis – a cohort study
Source: Arthritis Res Ther. 2017 Jan 13;19:5. doi: 10.1186/s13075-016-1208-6 (PMC5237153; doi:10.1186/s13075-016-1208-6)
Supplement: Additional file 4: — Prediction of escalation of therapy - univariate analyses (with and without stratification for incident vs. prevalent disease, and low vs. moderate to high disease activity). (DOCX 21 kb) [file 13075_2016_1208_MOESM4_ESM.docx]

**Additional file 4**

**Prediction of escalation of therapy - univariate analyses (with and without stratification for incident vs. prevalent disease, and low vs. moderate to high disease activity)**

| *Overall* | *(N = 185, N of events = 83)* |  |  |
| --- | --- | --- | --- |
| **Predictor** | **OR (95% CI)** | **p-value** | **AUC** |
| Previous DAS28 | 1.500 (1.348; 1.669) | **< 0.001** | 0.661 |
| Previous PDsynSS | 1.078 (1.013; 1.146) | **0.017** | 0.570 |
| Previous HAQ | 1.997 (1.311; 3.043) | **0.001** | 0.641 |
|  |  |  |  |
| *Incident* | *(N = 46, N of events = 26)* |  |  |
| Previous DAS28 | 1.811 (1.147; 2.860) | **0.011** | 0.721 |
| Previous PDsynSS | 1.037 (0.935; 1.149) | 0.494 | 0.569 |
| Previous HAQ | 2.188 (0.918; 5.212) | **0.077** | 0.669 |
|  |  |  |  |
| *Prevalent* | *(N = 139, N of events = 57)* |  |  |
| Previous DAS28 | 1.387 (1.098; 1.753) | **0.007** | 0.624 |
| Previous PDsynSS | 1.090 (1.008; 1.178) | **0.031** | 0.553 |
| Previous HAQ | 1.883 (1.161; 3.053) | **0.010** | 0.626 |
|  |  |  |  |
| *LDA* | *(N = 79, N of events = 24)* |  |  |
| Previous DAS28 | 1.173 (0.487; 2.826) | 0.721 | 0.520 |
| Previous PDsynSS | 0.895 (0.697; 1.149) | 0.385 | 0.532 |
| Previous HAQ | 0.986 (0.311; 3.126) | 0.980 | 0.452 |
|  |  |  |  |
| *nonLDA* | *(N = 106, N of events = 59)* |  |  |
| Previous DAS28 | 1.551 (1.008; 2.386) | **0.046** | 0.603 |
| Previous PDsynSS | 1.062 (0.994; 1.134) | 0.094 | 0.565 |
| Previous HAQ | 1.721 (0.979; 3.026) | 0.059 | 0.608 |

Escalation of therapy was defined as a new use or increased dose of glucocorticoids and/or DMARDS within 0-6 months after the measurement of U7S, DAS28, and HAQ (=at the same time or 6 months after the "previous" DAS28, PDsynUS, and HAQ).

PD=power doppler; syn = synovitis; SS=sum-score LDA = low disease activity (DAS28-CRP < 3.2)
