# Supplementary material for: The relationship between synovitis quantified by an ultrasound 7-joint inflammation score and physical disability in rheumatoid arthritis – a cohort study
Source: Arthritis Res Ther. 2017 Jan 13;19:5. doi: 10.1186/s13075-016-1208-6 (PMC5237153; doi:10.1186/s13075-016-1208-6)
Supplement: Additional file 5: — Prediction of escalation of therapy - multivariate analyses. (DOCX 24 kb) [file 13075_2016_1208_MOESM5_ESM.docx]

**Additional file 5**

**Prediction of escalation of therapy** - **multivariate analyses**

| *Overall* | *(N = 185, N of events = 83)* | |  |
| --- | --- | --- | --- |
| **Predictor** | **OR (95% CI)** | **p-value** | **AUC** |
| Previous PDsynSS | 1.025 (0.990; 1.062) | 0.474 | 0.661 |
| Previous DAS28 | 1.442 (1.163; 1.788) | **0.002** |  |
|  |  |  |  |
| *Incident RA* | *(N = 46, N of events = 26)* | |  |
| **Predictor** | **OR (95% CI)** | **p-value** | **AUC** |
| Previous PDsynSS | 0.973 (0.866; 1.094) | 0.648 | 0.729 |
| Previous DAS28 | 1.893 (1.150; 3.116) | **0.012** |  |
|  |  |  |  |
| *Prevalent RA* | *(N = 139, N of events = 57)* | |  |
| **Predictor** | **OR (95% CI)** | **p-value** | **AUC** |
| Previous PDsynSS | 1.047 (0.961; 1.095) | 0.293 | 0.611 |
| Previous DAS28 | 1.291 (0.996; 1.674) | 0.064 |  |

| *LDA* | *(N = 79, N of events = 24)* | |  |
| --- | --- | --- | --- |
| **Predictor** | **OR (95% CI)** | **p-value** | **AUC** |
| Previous PDsynSS | 0.879 (0.675; 1.146) | 0.341 | 0.545 |
| Previous DAS28 | 1.306 (0.522; 3.265) | 0.568 |  |
|  |  |  |  |
| *nonLDA* | *(N = 106, N of events = 59)* | |  |
| **Predictor** | **OR (95% CI)** | **p-value** | **AUC** |
| Previous PDsynSS | 1.040 (0.965; 1.121) | 0.305 | 0.613 |
| Previous DAS28 | 1.417 (0.892; 2.251) | 0.140 |  |

Escalation of therapy was defined as a new use or increased dose of glucocorticoids and/or DMARDS within 0-6 months after the measurement of U7S, DAS28, and HAQ (=at the same time or 6 months after the "previous" DAS28, PDsynUS, and HAQ).

PD=power doppler; syn = synovitis; SS=sum-score; LDA = low disease activity (DAS28-CRP < 3.2)

| *Overall* | *(N = 185, N of events = 83)* | |  |
| --- | --- | --- | --- |
| **Predictor** | **OR (95% CI)** | **p-value** | **AUC** |
| Previous PDsynSS | 1.054 (0.990; 1.123) | 0.102 | 0.643 |
| Previous HAQ | 1.817 (1.178; 2.803) | **0.007** |  |
|  |  |  |  |
| *Incident RA* | *(N = 46, N of events = 26)* | |  |
| **Predictor** | **OR (95% CI)** | **p-value** | **AUC** |
| Previous PDsynSS | 1.016 (0.915; 1.128) | 0.766 | 0.642 |
| Previous HAQ | 2.120 (0.874; 5.144) | 0.097 |  |
|  |  |  |  |
| *Prevalent RA* | *(N = 139, N of events = 57)* | |  |
| **Predictor** | **OR (95% CI)** | **p-value** | **AUC** |
| Previous PDsynSS | 1.066 (0.984; 1.155) | 0.116 | 0.619 |
| Previous HAQ | 1.686 (1.021; 2.785) | **0.041** |  |

| *LDA* | *(N = 79, N of events = 24)* | |  |
| --- | --- | --- | --- |
| **Predictor** | **OR (95% CI)** | **p-value** | **AUC** |
| Previous PDsynSS | 0.895 (0.696; 1.150) | 0.386 | 0.544 |
| Previous HAQ | 1.033 (0.322; 3.315) | 0.957 |  |
|  |  |  |  |
| *nonLDA* | *(N = 106, N of events = 59)* | |  |
| **Predictor** | **OR (95% CI)** | **p-value** | **AUC** |
| Previous PDsynSS | 1.056 (0.984; 1.133) | 0.131 | 0.625 |
| Previous HAQ | 1.653 (0.936; 2.919) | 0.083 |  |

Escalation of therapy was defined as a new use or increased dose of glucocorticoids and/or DMARDS within 0-6 months after the measurement of U7S, DAS28, and HAQ (=at the same time or 6 months after the "previous" DAS28, PDsynUS, and HAQ).

PD=power doppler; syn = synovitis; SS=sum-score; LDA = low disease activity (DAS28-CRP < 3.2)
