# Supplementary material for: The relationship between synovitis quantified by an ultrasound 7-joint inflammation score and physical disability in rheumatoid arthritis – a cohort study
Source: Arthritis Res Ther. 2017 Jan 13;19:5. doi: 10.1186/s13075-016-1208-6 (PMC5237153; doi:10.1186/s13075-016-1208-6)
Supplement: Additional file 6: — Comparison of baseline variables between groups of patients with one, two and three years of follow up. (DOCX 24 kb) [file 13075_2016_1208_MOESM6_ESM.docx]

Additional file 6

Comparison of baseline variables between groups of patients with one, two and three years of follow-up

| Variable | *Descriptive statistic* | One year FUP  *N = 70* | Two years FUP  *N = 59* | Three years FUP  *N = 54* | *p-value** |
| --- | --- | --- | --- | --- | --- |
| **Female** | *N (%)* | **52** (74.3 %) | **47** (79.7 %) | **43** (79.6 %) | *0.700* |
| **Age** | *mean (SD)* | **55.4** (14.5) | **55.0** (14.9) | **54.5** (12.4) | *0.850* |
|  | *median (95% CI)* | **55.6** (31.5; 75.4) | **58.1** (27.5; 72.4) | **55.9** (35.3; 73.0) |  |
| **Disease duration** | *mean (SD)* | **6.7** (7.9) | **5.9** (8.1) | **6.4** (7.4) | *0.264* |
|  | *median (95% CI)* | **4.0** (0.2; 26.7) | **2.9** (0.3; 23.1) | **3.8** (0.1; 22.3) |  |
| **Duration of symptoms** | *mean (SD)* | **6.5** (7.1) | **6.5** (8.1) | **6.2** (6.0) | *0.593* |
|  | *median (95% CI)* | **4.9** (0.3; 22.9) | **3.5** (0.3; 22.1) | **3.3** (0.9; 20.1) |  |
| **BMI** | *mean (SD)* | **27.0** (4.8) | **26.7** (4.9) | **26.3** (3.8) | *0.849* |
|  | *median (95% CI)* | **26.0** (19.8; 35.2) | **27.2** (19.8; 36.4) | **25.5** (21.3; 35.3) |  |
| **RF+ or ACPA+** | *N (%)* | **47** (68.1 %) | **38** (65.5 %) | **38** (70.4 %) | *0.859* |
| **Incident** | *N (%)* | **19** (27.1 %) | **17** (28.8 %) | **10** (18.5 %) | *0.400* |
| **Prevalent** | *N (%)* | **51** (72.9 %) | **42** (71.2 %) | **44** (81.5 %) |  |
| **Glucocorticoid use** | *N (%)* | **25** (35.7 %) | **29** (49.2 %) | **25** (46.3 %) | *0.264* |
| **csDMARD use** | *N (%)* | **59** (84.3 %) | **53** (89.8 %) | **48** (88.9 %) | *0.593* |
| **CRP** | *mean (SD)* | **7.5** (9.4) | **8.1** (9.5) | **6.8** (8.0) | *0.640* |
|  | *median (95% CI)* | **4.0** (0.5; 33.2) | **5.2** (0.2; 32.2) | **3.8** (0.6; 29.9) |  |
| **DAS28** | *mean (SD)* | **3.5** (1.5) | **3.5** (1.4) | **4.0** (1.5) | *0.892* |
|  | *median (95% CI)* | **3.2** (1.5; 6.1) | **3.6** (1.5; 6.2) | **4.1** (1.5; 6.1) |  |
| **GSsynSS** | *mean (SD)* | **6.1** (5.6) | **6.5** (6.0) | **8.3** (7.6) | *0.765* |
|  | *median (95% CI)* | **5.0** (0.0; 18.0) | **5.0** (0.0; 19.0) | **6.5** (0.0; 27.0) |  |
| **PDsynSS** | *mean (SD)* | **4.0** (4.7) | **3.3** (4.9) | **4.8** (6.2) | *0.152* |
|  | *median (95% CI)* | **2.0** (0.0; 16.0) | **2.0** (0.0; 12.0) | **3.0** (0.0; 21.0) |  |
| **GStenSS** | *mean (SD)* | **0.6** (1.1) | **0.7** (1.2) | **0.6** (1.1) | *0.977* |
|  | *median (95% CI)* | **0.0** (0.0; 3.0) | **0.0** (0.0; 3.0) | **0.0** (0.0; 3.0) |  |
| **PDtenSS** | *mean (SD)* | **0.8** (1.8) | **0.6** (1.5) | **0.6** (1.5) | *0.527* |
|  | *median (95% CI)* | **0.0** (0.0; 6.0) | **0.0** (0.0; 3.0) | **0.0** (0.0; 3.0) |  |
| **Erosions score** | *mean (SD)* | **1.1** (2.0) | **1.2** (2.1) | **1.2** (2.2) | *0.794* |
|  | *median (95% CI)* | **0.0** (0.0; 6.0) | **0.0** (0.0; 7.0) | **0.0** (0.0; 7.0) |  |
| **HAQ** | *mean (SD)* | **0.7** (0.8) | **0.8** (0.7) | **0.8** (0.7) | *0.648* |
|  | *median (95% CI)* | **0.5** (0.0; 2.4) | **0.5** (0.0; 2.3) | **0.8** (0.0; 2.1) |  |

**Pearson Chi-square test was used for categorical variables, and Kruskal-Wallis test for continuous variables.*

FUP=follow up. GS=gray scale; PD=power doppler; syn = synovitis, ten=tenosynovitis, ES= erosions score; SS=sum-score
